# Linking Cognitive Measures of Response Inhibition and Reward Sensitivity to Trait Impulsivity

**DOI:** 10.3389/fpsyg.2018.02306

**Published:** 2018-11-28

**Authors:** Ainara Jauregi, Klaus Kessler, Stefanie Hassel

**Affiliations:** ^1^Aston Brain Centre, School of Life and Health Sciences, Aston University, Birmingham, United Kingdom; ^2^Department of Psychiatry, Cumming School of Medicine, University of Calgary, Calgary, AB, Canada

**Keywords:** impulsivity, response inhibition, rapid-response, delay discounting, reward-delay

## Abstract

Impulsivity is regarded as a multifaceted construct that comprises two dimensions: rapid-response impulsivity and reward-delay impulsivity. It is unclear, however, which aspects of trait impulsivity, as assessed by self-report measures are related to rapid-response impulsivity and/or to reward-delay impulsivity, as different results have been reported in studies using both self-report and cognitive measures. This study aimed to directly relate self-report measures of impulsivity to cognitive measures of impulsivity in individuals at low- or high-levels on two impulsivity dimensions, specifically rapid-response impulsivity and reward-delay impulsivity. Participants were classified into high- or low-impulsivity groups based on (1) level of rapid-response impulsivity (determined by BIS-11 Motor subscale scores); (2) level of reward-delay impulsivity (determined by BIS/BAS subscale scores); and (3) a combination of rapid-response impulsivity and reward-delay impulsivity levels. Impulsivity was assessed using Go/No-Go, Stop-Signal and Delay-Discounting tasks and self-report measures. The high rapid-response impulsivity group showed significantly higher reward-delay impulsivity on both, the Delay-Discounting tasks and on self-report measures assessing reward-delay impulsivity, than the low-risk group. Based on the level of reward-delay impulsivity, the high reward-delay impulsivity group scored significantly higher on task-based (cognitive) and self-report measures assessing rapid-response inhibition than the low reward-delay impulsivity group. Combining both dimensions of impulsivity showed that the high-impulsivity group performed significantly worse in rapid-response paradigms and temporally discounted significantly more impulsively than the low-impulsivity group. Thus, combined impulsivity factors provide better assessment of impulsivity than each dimension alone. In conclusion, robust differences in impulsivity can be identified in non-clinical young adults.

## Introduction

Impulsivity, as a trait, is defined as “a predisposition toward rapid, unplanned reactions to internal or external stimuli without regard to the negative consequences of these reactions to the impulsive individual or to others” (Moeller et al., 2001; p. 1784). Thus, impulsivity is a socially relevant construct, impacting on society (e.g., business, criminal justice, education) as well as individuals (e.g., aggressive or anti-social behaviors) (Stanford et al., 2009). It has been reported to characterize several mental disorders, such as attention deficit/hyperactivity disorder (ADHD; Nigg, 2001), drug addiction (Jentsch and Taylor, 1999; Bari and Robbins, 2013), and bipolar spectrum disorders (Strakowski et al., 2009). Trait impulsivity is assessed by using self-report measures such as the Barratt Impulsiveness Scale (BIS-11, Patton and Stanford, 1995), the UPPS Impulsive Behavior Scale (UPPS; Whiteside and Lynam, 2001) or the Eysenck Personality Questionnaire (EPQ; Eysenck et al., 1985), specifically the Psychoticism subscale (Holt et al., 2003; Lewis et al., 2009).

Impulsivity is regarded as a multifaceted construct that comprises two dimensions: rapid-response impulsivity (also referred to as response inhibition or impulsive action) and reward-delay impulsivity or impulsive choice (Dawe et al., 2004; Dawe and Loxton, 2004; Winstanley et al., 2004; Alloy et al., 2009; Swann, 2010; MacKillop et al., 2016). Rapid-response impulsivity refers to a tendency to perform immediate actions, often without any forethought or a diminished ability to inhibit a pre-potent response (Moeller et al., 2001; Hamilton et al., 2015). Impulsive choice is described as diminished ability or willingness to tolerate delays (Hamilton et al., 2015). Although both constructs link back to core theoretical definitions of impulsivity they tend to correlate only weakly or not at all (Lane et al., 2003; Reynolds et al., 2006; Broos et al., 2012). These dimensions of impulsivity can be assessed using cognitive tasks, in addition to self-report measures. However, it is not clear which aspects of trait impulsivity, as assessed by self-report measures, such as the BIS-11, are related to response inhibition and/or to reward-delay impulsivity, as different results have been reported in studies using both self-report measures and cognitive tasks (Dick et al., 2010).

Rapid-response impulsivity has been investigated using the go/no-go task (GNGT) and the stop-signal task (SST) (Criaud and Boulinguez, 2013; Hamilton et al., 2015). These paradigms have been proposed to assess two different processes. GNGT assesses “action restraint” as it measures the inhibition of a planned response. Omission errors, i.e., withholding a response to a “Go” stimulus, and commission errors, i.e., responding to a “No Go” stimulus, index rapid-response impulsivity. SST measures “action cancelation” as it assesses the inhibition of an initiated response (Schachar et al., 2007; Eagle et al., 2008; Swick et al., 2011; Bari and Robbins, 2013). This distinction is supported by growing evidence from neuroimaging studies (e.g., Swick et al., 2011; Sebastian et al., 2013; Dambacher et al., 2014) which have shown different, in addition to common, neural patterns of activations when both paradigms are examined. The current study examined both paradigms to assess both functional aspects of impulsivity.

Self-report measures that tap into rapid-response impulsivity include the Motor-subscale of the BIS-11 and the Urgency subscale of the UPPS, the latter being reported to significantly correlate with SST performance (Wilbertz et al., 2014). However, significant associations between cognitive tasks assessing response inhibition and self-report measures are not consistently reported (e.g., Rodrìguez-Fornells et al., 2002; Horn et al., 2003; Spinella, 2004; Keilp et al., 2005; Lijffijt et al., 2005; Enticott et al., 2006; Reynolds et al., 2006; Aichert et al., 2012; Malesza and Ostaszewski, 2016).

The second dimension of impulsivity, reward-delay impulsivity or impulsive choice has been investigated in terms of the behavioural approach system (BAS) hypersensitivity model (e.g., Alloy and Abramson, 2010; Alloy et al., 2012; Molz et al., 2013; Duek et al., 2014; Nusslock et al., 2014). The reward hyper-sensitivity model proposes that individuals with a hyper-sensitive BAS may show exaggerated approach behaviors toward reward and goal cues (Molz et al., 2013). This can lead to drastic fluctuations of BAS activation and deactivation. Activation of the BAS by positive cues (e.g., positive life events, specifically those involving goal-striving and goal-attainment), can result in characteristics, or symptoms, such as increased energy, optimism, decreased need for sleep (Alloy et al., 2009). In contrast, deactivation of the BAS by negative cues (e.g., negative life-events, specifically those including failure to obtain – or loss of – goals/rewards) can result in depressive characteristics, or symptoms, resembling depressions, such as anhedonia, decreased energy or sadness (Urosević et al., 2008; Alloy and Abramson, 2010).

It has also been suggested that BAS hyperactivity may result in impulsive decision-making (Mason et al., 2012). When a reward cue activates the hyper-reactive BAS, anticipation of this reward may be responsible for generating an impulsive “state” (Bari and Robbins, 2013) which influences decision-making. BAS hyper-sensitivity toward rewards may result in an inability to delay gratification. This behavior is assessed using questionnaires such as the BIS/BAS scales (Carver and White, 1994). The UPPS (Lack of) Premeditation subscale has also been used to assess this inability to delay gratification (Lynam and Miller, 2004; Alloy et al., 2008, 2009; Urosević et al., 2008; Stojek et al., 2014). It has been reported that individuals scoring low on this subscale were more likely to prefer small and immediate rewards compared to larger but delayed rewards (Lynam and Miller, 2004; Stojek et al., 2014).

Previous experimental studies have assessed reward-delay impulsivity using the delay discounting task (DDT; Kirby et al., 1999; Johnson and Bickel, 2002). This task requires participants to choose between either small but immediate, or large but delayed rewards, typically amounts of hypothetical money. Higher rates of delay discounting are associated with self-report measures of impulsivity (e.g., Kirby et al., 1999), sensation seeking (Richards et al., 1999) or suicidal ideation and behavior (Cáceda et al., 2014). Greater discounting has also been reported in populations with impulse control problems, such as compulsive gamblers (Reynolds et al., 2006; Ledgerwood et al., 2009), acute alcohol, cocaine and methamphetamine users (Coffey et al., 2003; Bari and Robbins, 2013) or tobacco smokers (Baker et al., 2003). Similarly to inconsistent results reporting correspondence between cognitive tasks and self-report measures assessing rapid-response impulsivity, evidence shows that reward-delay tasks and self-report measures do not always correlate well (e.g., Reynolds et al., 2006; Murphy and MacKillop, 2012). Stojek et al. (2014), for instance, suggested that reward-delay tasks may not be sufficiently sensitive to assess particular impulsivity dimensions, as measured by questionnaires, yet, the reverse could also be true due to the biased nature of self-report measures.

The current study set out to re-investigate previous failures of finding a strong relationship between cognitive and self-report measures of impulsivity dimensions, specifically response inhibition and reward-delay. Therefore, it is of vital importance to investigate these two dimensions in conjunction. Here, we administered self-report measures of trait impulsivity to classify individual impulsivity levels, and then relate this classification to cognitive measures of impulsivity. The aim was to clarify the robustness of self-report questionnaires as genuine predictors of behavioral impulsivity. Although the two dimensions of impulsivity, rapid-response and reward-delay impulsivity, have been extensively investigated, there are still considerable issues that need to be clarified: First, the lack of consistency regarding the extent of associations between cognitive tasks and self-reports; and secondly, the need to further understand the relationship between these two dimensions of impulsivity. In the present study, associations between rapid-response impulsivity and reward delay impulsivity will be investigated in an undergraduate student population. Inhibitory control and reward sensitivity seem naturally inter-related concepts, however, studies of such interactions have been limited. Contrary to most published reports, Meda et al. (2009) showed good correspondence between cognitive and self-report measures of impulsivity, including a delay-discounting task and BIS/BAS, BIS-11 measures.

Here, we classified participants into high and low impulsive groups, depending on the absence, or presence, of the following: (a) level of rapid-response impulsivity – as determined by BIS-11 Motor sub-scale scores, (b) level of reward-delay impulsivity – as determined by BAS Total scores, and (c) a combination of both levels. Factor analyses of the impulsivity construct have proposed that impulsivity domains such as rapid-response impulsivity and reward-delay impulsivity reflect discrete impulsivity dimensions (e.g., MacKillop et al., 2016). However, overlap has also been reported (e.g., Meda et al., 2009), suggesting that impulsivity domains may be less distinct. A combined group was therefore added to investigate to what extent trait dimensions of rapid-response impulsivity and reward-delay impulsivity together would impact on and interact with cognitive tasks. These groups performed response inhibition and reward-delay experimental tasks to clarify the relationship between self-report measures and behavioral effects, and to assess how sensitive these measures are to differences between high- and low-impulsivity groups. Associations between our cognitive tasks assessing rapid-response and reward-delay impulsivity will also be examined to further elucidate the contradictory results reported to date. Overall, this will help clarifying the relationship between self-report and cognitive measures of trait impulsivity as well as the relationship between the dimensions of response-inhibition and reward delay.

We hypothesized that: (1) Performance on the cognitive tasks assessing each of the two dimensions of impulsivity (i.e., rapid-response and reward-delay impulsivity) will correlate with the corresponding self-report measures assessing each dimension. (2) Single high-impulsive vs. single low-impulsive and combined high impulsive vs. low-impulsive groups will perform significantly differently on cognitive tasks assessing impulsivity: High-impulsive individuals (single and combined high-risk) are expected to show impaired response inhibition on the GNGT and SST, and an inability to delay reward on the DDT. (3) Single high-impulsive vs. single low-impulsive and combined high- vs. low-impulsive groups will score significantly differently on self-report measures assessing rapid-response and reward-delay impulsivity: High-impulsive individuals (single and combined impulsivity dimensions) are expected to score significantly higher on self-report measures than the low-impulsive group.

## Materials and Methods

### Participants

Participants were recruited through advertisements around Aston University. 175 undergraduate students who were aged 18 to 26 and enrolled in psychology or business courses at Aston University participated and received either course credit or £7.50 for their time. Eight participants were excluded from the analysis due to incomplete data, resulting in a total of 167 participants for analysis (143 females, Mage = 19.43, *SD*age = 1.72). Our sample was ethnically diverse, consisting of White (40%), Asian (37%), Black (13%), Chinese (5%), and Mixed (7%) heritage. This is in line with the ethnic representation of the West Midlands and Birmingham, United Kingdom, region.

### Procedure

The experimental procedures were in accordance with the Declaration of Helsinki and approved by the Aston University Ethics Committee, including consent, in writing, prior to any data collection. During each session, which lasted approximately 60 min, each participant completed the cognitive tasks measuring rapid-response impulsivity and reward-delay sensitivity in a randomized order. All tasks were administered using EPrime 2.0 Professional (Psychology Software Tools^1^). Demographic and self-report questionnaires were completed in randomized order via Bristol Online Surveys^2^, using the same computer. A researcher was present during all data collection. Cognitive tasks and self-report measures were presented in a counter-balanced order.

### Cognitive Tasks

#### Rapid-Response Impulsivity: Go/No-Go Task (GNGT)

**FIGURE 1:**
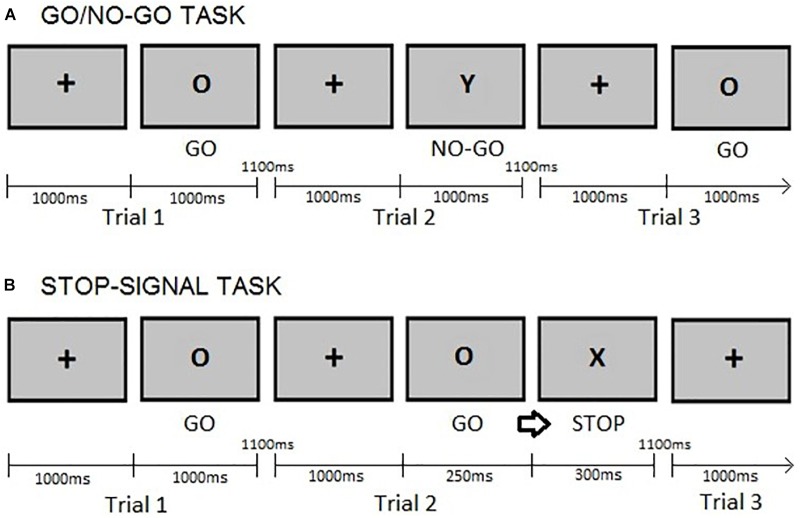
Example of trials: **(A)** Go/no-go task. Participants provided a response as fast as possible to a “go” (letter O) stimulus by pressing a button on a keyboard but refrained from reacting to a “no-go” (letter Y) stimulus. **(B)** Stop-signal task. Participants were requested to withhold their response (‘stop’) when the go cue (letter O) was followed by a stop-signal (letter X). In both tasks, the fixation cross is presented for 1000ms and the go cues for a maximum of 1000ms or until a response is given. The inter-stimulus interval is 1100ms as in Rubia et al. (2001). The stop-signal delay (SSD) was set at 250ms after the presentation of the go cue and the stop cue lasted for 300ms, as in Dambacher et al. (2014).

This study used a modified version of the GNGT described by Rubia et al. (2001), presenting 75% go trials instead of 70% go trials. This decision was based on previous studies (e.g., Zheng et al., 2008), which argued that using a simpler go/nogo task, i.e., one with a lower percentage of no-go trials, would reduce the load of other cognitive functions, e.g., working memory processes, apart from action restraint. The stimuli used in this GNGT were letters. Participants first completed a practice block of 20 trials, the proceeded to complete two blocks of 140 trials each, with the option of taking a rest between blocks. The variables of interest were the reaction times (GNGT RTs) on correct responses to “go” trials and the raw number of commission errors during “no-go” trials. For more detail on the task, please see Figure 1A. GNGT have been reported to demonstrate content validity (Moeller et al., 2001), concurrent validity – evidenced by correlations with measures such as the SST and moderate to high level of test-retest reliability (Weafer et al., 2013; Hamilton et al., 2015).

#### Rapid-Response Impulsivity: Stop-Signal Task (SST)

In this paradigm, a “go” stimulus was occasionally followed by a “stop signal” (delayed by 250ms) at an occurrence rate of 25% of the total trials, see Figure 1B for a description of the SST and its timing parameters. Participants completed a practice block of 20 trials and two blocks of 140 trials each, having a rest between blocks. The variables of interest were the Stop-Signal Task reaction times (SSRTs) on “go” trials and of the raw number of commission errors on “stop” trials. The latter was included because the proportion of successful stop trials (calculated as the number of responses made on stop-trials divided by the total number of stop trials) provides information about the ability to withhold an already initiated response (Dougherty et al., 2005). SSRTs were estimated by subtracting the stop-signal delay (250ms) from the mean “go”-trial reaction time, a procedure following that of Logan et al. (1997). SSTs have been reported to demonstrate good content validity (Moeller et al., 2001), good concurrent validity – evidenced by correlations with measures such as the GNG, and moderately high test-retest reliability (Weafer et al., 2013; Wostmann et al., 2013; Hamilton et al., 2015).

#### Reward-Delay Impulsivity: Delay Discounting Task (DDT)

The DDT (Kirby et al., 1999) consisted of 27 hypothetical choices between a small but immediate reward (£11–£80) and a larger reward (£25–£85) delayed between 7 and 186 days. There was a 1000ms pause between sentences. The primary measure of delay discounting was the proportion of the smaller, immediate rewards selected out of the 27 choices. The most widely used strategy to calculate the discounting rate is based on either a hyperbolic or an exponential function (e.g., Mazur, 1987; Coffey et al., 2003; Dougherty et al., 2014). However, the proportion score has also been used previously to assess delay discounting (e.g., Ersner-Hershfield et al., 2008; Magen et al., 2008; Benningfield et al., 2014). This alternative is a more straight-forward approach, as it does not make assumptions about the shape of the discounting curve and has similar results to the hyperbolic model (Benningfield et al., 2014), hence we decided to employ this alternative for the current study. Previous research has demonstrated that delay-discounting is stable over time (Simpson and Vuchinich, 2000; Ohmura et al., 2006; Shamosh et al., 2008) and internally consistent (Cronbach’s α = 0.89) (Hurst et al., 2011). Construct validity for the DDT has been shown by significant correlations with neurocognitive measures of impulsivity (Perales et al., 2009) and decision making (Monterosso et al., 2001).

### Self-Report Measures

#### Barratt Impulsiveness Scale

The BIS-11 (Patton and Stanford, 1995) is a well validated and reliable measure of trait impulsivity (Stanford et al., 2009). It consists of 30 items, comprising three subscales: non-planning (lack of future sense, 11 items), motor (acting on the spur of the moment without thinking, 11 items), attentional (distractibility, lack of sustained attention, 8 items), rated on a four-point Likert scale (1 = rarely/never, 2 = occasionally, 3 = often, 4 = almost always/always). Stanford et al. (2009) reported that BIS-11 total scores demonstrate reasonable test–retest reliability over 1 month (Spearman’s ρ = 0.83) and good internal consistency (α = 0.83), a finding similar to that of DeVellis (1991), who reported an α = 0.82. High scores in the sum of all subscales of the BIS-11 indicate high levels of trait impulsivity as a heterogeneous concept, while high scores on each specific subscale describe which components of impulsivity have a heavier weight.

#### Eysenck Personality Questionnaire

The EPQ (Eysenck et al., 1985) is 48-item questionnaire (responses are YES or NO) with four subscales: Psychoticism, with items related to impulsivity; Extraversion, which relates to sociability and venturesomeness; Neuroticism, which measures emotional stability, and a Lie scale assessing defensiveness. Higher values on each subscale indicate higher levels of the corresponding personality trait. The EPQ shows good/acceptable internal consistency; α ranging from 0.7 (for the Lie scale) to 0.9 (Extraversion and Neuroticism) (Corulla, 1987; Muniz et al., 2005). Cronbach’s α for the traditional dichotomous format shows good reliability for the three subscales of the EPI: psychoticism sub-scale α = 0.77, extra-version subscale α = 0.78 and neuroticism subscale α = 0.7 (Muniz et al., 2005). This self-report measure, and similar versions of Eysenck’s personality questionnaires, have previously been used to investigate the association between impulsivity self-reports and cognitive tasks (e.g., Logan et al., 1997; Marsh et al., 2002), but yielded inconsistent results. Adding this measure allowed further exploration of impulsivity dimensions.

#### UPPS Behavior Scale

The UPPS (Whiteside and Lynam, 2001) is a 44-item, Likert-type scale (from 1 = strongly disagree to 4 = strongly agree) which has four factor analytically based subscales: Urgency, (lack of) Premeditation, (lack of) Perseverance and Sensation Seeking. The sub-scales have good convergent validity, good discriminant validity and internal consistency of α = 0.8 or greater for each of the subscales (e.g., Cyders and Smith, 2007; Smith et al., 2007; Cyders, 2013). High scores on this questionnaire indicate high levels of trait impulsivity. This scale was included as an additional measure of the two dimensions explored here, because it includes one specific subscale for each dimension (Urgency – for rapid-response impulsivity and (Lack of) Premeditation – for reward-delay). Furthermore, Wilbertz et al. (2014) found the Urgency subscale, rather than the BIS-11, to be the only measure that explained individual variability on response inhibition performance. Hence, this measure might help clarifying how strong the relationships are between the two impulsivity dimensions investigated here.

#### Behavioral Inhibition System/Behavioral Activation System Scales

The Behavioral Inhibition System/Behavioral Activation System scales (BIS/BAS; Carver and White, 1994) consist of 20 items that are rated on a 4-point Likert-type scale (from 1 = strongly disagree to 4 = strongly agree) and comprise three BAS subscales (reward responsiveness, drive and fun seeking) and one BIS subscale (reactions to the expectation of punishment). These scales assessed participants’ sensitivity of the BAS and the behavioural inhibition system (BIS) to positive and negative cues. The BIS/BAS scales show good internal validity, with Cronbach’s α for BAS-Reward, BAS-Drive and BAS-Fun being 0.79, 0.61, 0.68, respectively (Franken et al. (2005) or 0.69, 0.82, 0.76, respectively (Gomez et al., 2005) and 0.81 to 0.83 for BAS (total) (Jorm et al., 1999; Erdle and Rushton, 2010). For the BIS scale internal validity was modest to good with Cronbach’s α ranging from 0.59 (Franken et al. (2005) to 0.71 (Erdle and Rushton, 2010) or 0.76 (Jorm et al., 1999) or 0.82 (Gomez et al., 2005). High scores on this self-report measure indicate high sensitivity to the BAS or BIS system (see the Introduction for further details). High scores in the sum of all subscales of the BAS subscales, BAS Total, indicate high levels of BAS sensitivity, while high scores on each specific subscale describe which components of BAS sensitivity have a heavier weight (reward responsiveness, drive, and fun seeking).

#### Group Assignment

For statistical analyses, participants were assessed based on (1) rapid response impulsivity; (2) reward delay impulsivity; and (3) a combination of rapid response impulsivity and reward delay impulsivity. High and low impulsivity group assignment was based on:

For (1) Their BIS-11 Motor sub-scale scores: To assess rapid-response impulsivity, high- and low-impulsive individuals were selected from the sample using the highest 35th percentile and the lowest 35th percentile, respectively. This procedure is in line with that of Wilbertz et al. (2014) and resulted in two extreme groups, i.e., a high rapid-response impulsivity group and a low rapid-response impulsivity group.

For (2) Their BAS Total scores: To assess reward-delay impulsivity, high-impulsive individuals were selected from the sample using the highest 15th percentile on the BAS Total scale. This resulted in a high reward-delay impulsivity group. For the low reward-delay impulsivity group moderate BAS scores (between the 40th and 60th percentiles) were chosen. The reason for choosing the moderate BAS score and not the low BAS score is because low BAS scores have previously been linked with unipolar depression (Fowles, 1988; Depue and Iacono, 1989; Kasch et al., 2002) as well as excessively decreased goal-directed activity, loss of interest and anhedonia (Alloy et al., 2012). Additionally, a moderate BAS score is closer to the mean on the BAS sensitivity dimension thus representing a more normalized statistical perspective. This procedure in line with that of Alloy et al. (2012) and resulted in a low reward-delay impulsivity group.

For (3) A combination of rapid-response impulsivity and reward-delay impulsivity: Here, participants whose scores fulfilled both of the above criteria were included. The high-impulsivity group consisted of participants scoring within the highest 35th percentile of the BIS-11 Motor sub-scale and within the highest 15th percentile on the BAS-Total scale. The low-impulsivity group included participants scoring below 35% on the BIS-11 Motor sub-scale and between the 40th and 60th percentiles on the BAS-Total scale.

### Statistical Analysis

The Kolmogorov–Smirnov test for normality and Levene’s test for homogeneity of variances were used to assess whether assumptions were met. Spearman’s ρ was used to examine correlations between self-report measures and cognitive tasks as a first, exploratory step. Subsequently, multivariate analyses of covariance (MANCOVA) were conducted to test differences between groups on the self-report measures and cognitive tasks separately, instead of multiple ANCOVAs or a single MANCOVA on both self-report measures and cognitive tasks, to reduce the chance of committing a Type I error. In cases where MANCOVAs were not significant, univariate ANOVAs were conducted to test *a priori* hypotheses, using Bonferroni correction as implemented in SPSS, which adjusts the *p*-value. Variables included in the analyses were the cognitive tasks measures (see above) and the scores on the self-report measures along with a covariate, participants’ history of mental health (as assessed by past or current visits to a mental health provider). Sex or age did not meet all the assumptions to be included as a covariate, i.e., independence of covariate and independent variable, homogeneity of the regression slopes and, when included, did not change the result of the analyses. Partial eta-squared (η^2^) values were calculated to measure effect size and interpreted using Cohen’s (Cohen, 1988) guidelines for determining small (0.01), medium (0.06) and large (0.14) effects. All statistical analyses were performed in SPSS (Version 22.0; SPSS Inc., Chicago, IL, United States).

## Results

### Correlational Analyses

As some of the variables did not meet assumptions for parametric analysis, Spearman’s ρ was used for all analyses to examine the correlations between the variables from the self-report measures and the cognitive tasks. The significance level was adjusted using Bonferroni correction, so that significant correlations reported here are based on the adjusted *p*-values (see Table 1). Significant positive correlations were observed between the DDT and the BIS-11 Total score rs = 0.28 (*p* = 0.000, 2-tailed) and BIS-11 Non-Planning subscale rs = 0.29 (*p* = 0.000, 2-tailed). Similarly, the self-report measures assessing different aspects of impulsivity, and their subscales, also showed significant correlations: The BIS-11 Total score and the three BIS-11 subscales (motor, attention and non-planning) significantly correlated with all UPPS subscales (urgency, premeditation and perseverance) except for Sensation Seeking (which significantly correlated with the BIS-11 Total score only), with the EPQ Psychoticism subscale and BAS Fun subscale, while the BIS-11 Motor subscale also significantly correlated with BAS Drive. The BAS Reward subscale significantly correlated with two UPPS subscales, (Lack of) Perseverance and (Lack of) Premeditation, while the BAS Drive subscale significantly correlated with (Lack of) Perseverance. The BAS Fun subscale significantly correlated with the Urgency, (Lack of) Premeditation and Sensation Seeking UPPS subscales (for all coefficients, see Table 1).

**Table 1:** Correlational analyses between impulsivity variables using Spearman’s ρ; Bonferroni adjusted *p*-value: 0.05/17 = 0.002.

Variable	1	2	3	4	5	6	7	8	9	10	11	12	13	14	15	16	17
(1) GNGT number errors	1	−0.25^∗^	0.29^∗^	−0.32^∗^	0.18	0.17	0.19	0.14	0.11	0.03	−0.05	0.01	0.07	0.09	0.09	0.07	0.03
(2) GNGT RT		1	−0.05	0.24^∗^	−0.01	0.01	−0.02	0.01	0.01	0.04	0.15	−0.02	0.00	0.10	−0.04	−0.08	−0.10
(3) SST number errors			1	−0.71^∗^	0.03	0.10	0.06	0.12	0.10	0.02	−0.03	−0.02	0.05	0.13	0.04	−0.01	0.03
(4) SSRT				1	−0.04	−0.06	−0.04	−0.09	−0.03	0.04	0.10	0.01	−0.07	−0.02	−0.04	0.01	−0.08
(5) DDT					1	0.28^∗^	0.29^∗^	0.16	0.21	0.09	0.06	0.07	0.13	0.07	0.09	0.04	0.06
(6) BIS-11 total						1	0.89^∗^	0.72^∗^	0.85^∗^	0.03	0.14	0.44^∗^	0.31^∗^	0.54^∗^	0.61^∗^	0.49^∗^	0.17^∗^
(7) BIS-11 non-planning							1	0.47^∗^	0.62^∗^	−0.08	0.02	0.28^∗^	0.25^∗^	0.47^∗^	0.65^∗^	0.53^∗^	0.09
(8) BIS-11 attention								1	0.52^∗^	0.09	0.06	0.31^∗^	0.23^∗^	0.49^∗^	0.36^∗^	0.40^∗^	0.19
(9) BIS-11 motor									1	0.13	0.29^∗^	0.51^∗^	0.26^∗^	0.43^∗^	0.49^∗^	0.26^∗^	0.18
(10) BAS reward										1	0.34^∗^	0.34^∗^	−0.12	−0.03	−0.33^∗^	−0.31^∗^	0.20
(11) BAS drive											1	0.42^∗^	−0.03	0.02	−0.05	−0.33^∗^	0.18
(12) BAS fun												1	0.23	0.26^∗^	0.24^∗^	0.08	0.51^∗^
(13) EPQ psychoticism													1	0.12	0.16	0.21	0.15
(14) UPPS urgency														1	0.39^∗^	0.41^∗^	0.06
(15) UPPS premeditation															1	0.48^∗^	0.09
(16) UPPS perseverance																1	−0.03
(17) UPPS sensation seeking																	1

*GNGT, Go/No-Go Task; RT, reaction time; SST, Stop-Signal Task; SSRT, Stop-Signal reaction time; DDT, Delay Discounting Task; BIS-11, Barratt Impulsiveness Questionnaire 11; BAS, Behavioral Activation System; EPQ, Eysenck Personality Questionnaire; UPPS, Urgency, Premeditation Perseverance, Sensation (Questionnaire). ^∗^p < 0.002 (2-tailed).*

### Cognitive Tasks and Questionnaire Results Based on Impulsivity Group

#### Rapid-Response Impulsivity

Rapid-response impulsivity was assessed by computing the scores of the BIS-11 Motor subscale. Those with scores within the highest 35th percentile of the motor scores in the whole sample constitute a high rapid-response impulsivity group (*n* = 73). Participants within lowest 35th percentile on the BIS-11 Motor subscale constitute a low rapid-response impulsivity group (*n* = 65). This procedure is in line with Wilbertz et al. (2014). Means and standard deviations of variables are presented in Tables 2, 3.

**Table 2:** MANCOVA results for cognitive measures for *Rapid-response impulsivity group*: Presented are raw means (standard deviations) and *F*-statistic.

Variable	Low Rapid-response impulsivity group (*n* = 65)	High Rapid-response impulsivity group (*n* = 73)	*F*(1,135)	Partial Eta Squared
GNGT – number of commission errors	5.17 (6.3)	5.71 (5.6)	0.27	0.00
GNGT – Mean RT	363.19 (55.4)	358.96 (64.8)	0.18	0.00
SST – number of commission errors	12.91 (9.1)	15.03 (9.9)	1.7	0.01
SSRT	275.19 (160.4)	258.89 (162.9)	0.41	0.00
DDT	0.53 (0.2)	0.59 (0.1)	7.56^∗∗^	0.05

*For the DDT, the proportion of small-immediate/large-delayed reward choices is shown. GNGT, Go/No-Go Task; RT, reaction time; SST, Stop-Signal Task; SSRT, Stop-Signal reaction time; DDT, Delay Discounting Task. ^∗∗^p < 0.01.*

**Table 3:** MANCOVA results for psychometric measures for *Rapid-response impulsivity group*: Presented are raw means (standard deviations) and *F*-statistic.

Variable	Low Rapid-response impulsivity group (*n* = 65)	High Rapid-response impulsivity group (*n* = 73)	*F*(1,135)	Partial Eta Squared
BAS total	39.02 (4.1)	41.41 (4.8)	9.86^∗∗^	0.07
BAS reward	17.51 (1.8)	17.75 (1.9)	0.66	0.01
BAS drive	10.68 (1.9)	11.21 (2.4)	2.01	0.02
BAS fun	10.83 (1.8)	12.45 (2.1)	23.56^∗∗^	0.15
EPQ psychoticism	2.00 (1.5)	2.89 (1.7)	10.21^∗∗^	0.07
UPPS urgency	26.17 (5.3)	31.93 (6.9)	32.14^∗∗^	0.19
UPPS (lack of) premeditation	18.02 (3.6)	22.92 (5.3)	41.05^∗∗^	0.23
UPPS (lack of) perseverance	18.57 (4.9)	22.12 (5.2)	17.41^∗∗^	0.11
UPPS sensation seeking	32.09 (7.1)	33.82 (7.0)	2.12	0.02

*BAS, Behavioral Activation System; EPQ, Eysenck Personality Questionnaire; UPPS, Urgency, Premeditation Perseverance, Sensation (Questionnaire). ^∗∗^p < 0.01.*

A MANCOVA was performed to contrast the two groups on the cognitive tasks while controlling for mental health history (see Table 2). The overall MANCOVA showed that groups classified by their level of motor impulsivity were not significantly different from each other (Wilks λ = 0.94, *F*(5,131) = 1.77, *p* = 0.123, η^2^ = 0.06). Although the overall MANCOVA was not significant, univariate ANOVAs were conducted to test *a priori* hypotheses, using Bonferroni correction as implemented in SPSS. This revealed significant differences between groups for the proportion of smaller vs. larger reward choices in the DDT, with a close to medium effect size estimate (*F*(1,135) = 7.56, *p* = 0.007, η^2^ = 0.05).

A MANCOVA was performed to contrast the two groups on the self-report measures while controlling for mental health history (see Table 3). The overall MANCOVA showed that groups classified by their level of motor impulsivity were significantly different from each other (Wilks λ = 0.62, *F*(8,128) = 9.84, *p* = 0.000, η^2^ = 0.38). Univariate ANOVAs revealed significant differences between groups for BAS Total scores (*F*(1,135) = 9.86, *p* = 0.002, η^2^ = 0.07), BAS Fun Seeking scores (*F*(1,135) = 23.56, *p* = 0.000, η^2^ = 0.15), EPQ Psychoticism scores (*F*(1,135) = 10.21, *p* = 0.002, η^2^ = 0.07), UPPS Urgency (*F*(1,135) = 32.14, *p* = 0.000, η^2^ = 0.19), UPPS (Lack of) Premeditation scores (*F*(1,135) = 41.05, *p* = 0.000, η^2^ = 0.23) and UPPS (Lack of) Perseverance scores (*F*(1,135) = 17.41, *p* = 0.000, η^2^ = 0.11). Partial eta-squared estimates suggested medium to large effect sizes for these measures, ranging from 0.07 to 0.23.

#### Reward-Delay Impulsivity

Reward-delay impulsivity was assessed by computing the BAS Total (BAS-T) scores (as in Alloy et al., 2012). Participants with scores in the highest 15th percentile on the BAS-T (high BAS-T score cut point ≥ 45) were assigned to a high reward-delay impulsivity group (*n* = 30). The low reward-delay impulsivity group (*n* = 53) consisted of participants with moderate scores, i.e., 40th and 60th percentiles, on the BAS (cut points ≥ 38 and ≤ 41). Means and standard deviations of variables are presented in Tables 4, 5.

**Table 4:** MANCOVA results for cognitive measures for *Reward-delay impulsivity group*: Presented are raw means (standard deviations) and *F*-statistic.

Variable	Low Reward-delay impulsivity group (*n* = 53)	High Reward-delay impulsivity group (*n* = 30)	*F*(1,80)	Partial Eta Squared
GNGT – number of commission errors	3.47 (3.5)	5.07 (4.2)	3.57	0.04
GNGT – Mean RT	359.10 (53.8)	378.91 (70.5)	2.44	0.03
SST – number of commission errors	13.68 (9.5)	15.07 (10.6)	0.33	0.00
SSRT	260.90 (174.1)	279.30 (165.9)	0.34	0.00
DDT	0.53 (0.1)	0.60 (0.1)	4.42^∗^	0.05

*For the DDT, the proportion of small-immediate/large-delayed reward choices is shown. GNGT, Go/No-Go Task; RT, reaction time; SST, Stop-Signal Task; SSRT, Stop-Signal reaction time; DDT, Delay Discounting Task. ^∗^p < 0.05.*

**Table 5:** MANCOVA results for psychometric measures for *Reward-delay impulsivity group*: Presented are raw Means (standard deviations) and *F*-statistic.

Variable	Low Reward-delay impulsivity group (*n* = 53)	High Reward-delay impulsivity group (*n* = 30)	*F*(1,80)	Partial Eta Squared
BIS-11 total	61.46 (11.6)	69.42 (12.5)	9.08^∗∗^	0.10
BIS-11 non-planning	22.25 (4.8)	24.64 (5.4)	4.39^∗^	0.05
BIS-11 attention	18.51 (4.7)	19.25 (4.1)	0.86	0.01
BIS-11 motor	21.33 (4.9)	25.83 (4.7)	16.97^∗∗^	0.18
EPQ psychoticism	2.17 (1.5)	2.83 (1.7)	3.58	0.04
UPPS urgency	29.19 (6.7)	31.17 (6.8)	2.24	0.03
UPPS (lack of) premeditation	20.42 (4.4)	20.80 (6.0)	0.21	0.03
UPPS (lack of) perseverance	20.89 (5.1)	18.27 (4.6)	5.03^∗^	0.06
UPPS sensation seeking	31.32 (6.9)	36.90 (7.7)	11.32^∗∗^	0.12

*BIS-11, Barratt Impulsiveness Questionnaire 11; EPQ, Eysenck Personality Questionnaire; UPPS, Urgency, Premeditation Perseverance, Sensation (Questionnaire). ^∗^p < 0.05, ^∗∗^p < 0.01.*

A MANCOVA was performed to contrast the low reward-delay impulsivity group and the high reward-delay impulsivity group on all cognitive measures’ variables (see Table 4), controlling for mental health history. The overall MANCOVA showed that the groups divided by their level of reward-delay impulsivity were significantly different (Wilks λ = 0.87, *F*(5,76) = 2.36, *p* = 0.048, η^2^ = 0.13). Univariate ANOVAs were conducted to test *a priori* hypotheses, using Bonferroni correction as implemented in SPSS, which revealed significant differences between groups for the proportion of smaller vs. larger reward choices in the DDT, with a close to medium effect size estimate (*F*(1,80) = 4.42, *p* = 0.039, η^2^ = 0.05).

A MANCOVA was performed to contrast the low reward-delay impulsivity group and the high reward-delay impulsivity group on all self-report measures’ variables (see Table 5), controlling for mental health history. The overall MANCOVA showed that the groups divided by their level of reward-delay impulsivity were significantly different (Wilks λ = 0.55, *F*(9,72) = 6.44, *p* = 0.000, η^2^ = 0.45). Univariate ANOVAs revealed significant differences between groups for BIS-11 Total scores (*F*(1,80) = 9.08, *p* = 0.003, η^2^ = 0.10), BIS-11 Non-Planning scores (*F*(1,80) = 4.39, *p* = 0.039, η^2^ = 0.05), BIS-11 Motor scores (*F*(1,80) = 16.97, *p* = 0.000, η^2^ = 0.18), UPPS (Lack of) Perseverance scores (*F*(1,80) = 5.03, *p* = 0.028, η^2^ = 0.06) and UPPS Sensation seeking scores (*F*(1,80) = 11.32, *p* = 0.001, η^2^ = 0.12). Partial eta-squared estimates suggested medium to large effect sizes for these measures, ranging from 0.05 to 0.18.

#### Rapid-Response Impulsivity and Reward-Delay Impulsivity Combined Group

Here, groups divided by their level of rapid-response impulsivity and reward-delay impulsivity were combined into either a single high-impulsivity group or a single low-impulsivity group, using the combined scores of the BIS-11 Motor and BAS-Total scales. The high-impulsivity group (*n* = 23) consisted of participants scoring high on both scales (scores within the highest 35th percentile of the BIS-11 Motor subscale in the whole sample and the highest 15th percentile of BAS-Total scale). The low-impulsivity group (*n* = 22) included participants scoring low on the BIS-11 Motor subscale (lowest 35th percentile) and moderately on the BAS-Total scale (between the 40th and 60th percentiles). Means and standard deviations of variables are presented in Table 6.

**Table 6:** MANCOVA results for behavioral measures for *Rapid-response impulsivity and Reward-delay impulsivity combined group*: Presented are raw Means (standard deviations) and *F*-statistic.

Variable	Low impulsivity group (*n* = 22)	High impulsivity group (*n* = 23)	*F*(1,42)	Partial Eta Squared
GNGT – number of commission errors	2.50 (2.3)	5.17 (4.5)	7.50^∗∗^	0.15
GNGT – Mean RT	357.01 (51.5)	388.19 (76.6)	2.74	0.6
SST – number of commission errors	9.73 (7.1)	15.91 (10.3)	5.95^∗^	0.12
SSRT	307.99 (173.7)	279.86 (147.4)	0.38	0.01
DDT	0.49 (0.2)	0.62 (0.1)	8.76^∗∗^	0.17

*For the DDT, the proportion of small-immediate/large-delayed reward choices is shown. GNGT, Go/No-Go Task; RT, reaction time; SST, Stop-Signal Task; SSRT, Stop-Signal reaction time; DDT, Delay Discounting Task. ^∗^p < 0.05, ^∗∗^p < 0.01.*

A MANCOVA was performed to contrast the two groups on all the cognitive measures of impulsivity (see Table 6), controlling for mental health history. The overall MANCOVA showed that the groups were significantly different (Wilks λ = 0.64, *F*(5,38) = 4.28, *p* = 0.003, η^2^ = 0.36). Univariate ANOVAs revealed significant differences between groups for GNGT raw number of commission errors (*F*(1,42) = 7.50, *p* = 0.009, η^2^ = 0.15), SST raw number of commission errors (*F*(1,42) = 5.95, *p* = 0.019, η^2^ = 0.12) and the proportion of smaller vs. larger reward choices in the DDT (*F*(1,42) = 8.76, *p* = 0.005, η^2^ = 0.17). Partial eta-squared estimates suggested large effect sizes for these measures, ranging from 0.13 to 0.17.

## Discussion

In this study, we compared different cognitive and self-report measures of impulsivity within a sample of undergraduate students. We examined how sensitive these measures are to detect differences between groups classified as being low in impulsivity and high in impulsivity, based on two factors: level of rapid-response impulsivity (Swann et al., 2009) and reward-delay impulsivity (Alloy et al., 2006). The results comparing groups via general linear model analyses partly supported our three hypotheses: (1) The group with elevated rapid-response impulsivity had significantly higher scores on self-reports measuring reward-delay impulsivity than the low rapid-response impulsivity group. The group with elevated reward-delay impulsivity had significantly higher scores on self-reports measuring rapid-response impulsivity than the low reward-delay impulsivity group. (2) The high-impulsivity group, as defined by high scores on rapid-response impulsivity and reward-delay impulsivity self-report measures, performed significantly worse on the GNGT and SST and, on the DDT, preferred small but immediate rewards over larger, delayed rewards significantly more often than the low-impulsivity group. (3) Correlations between the cognitive tasks and self-report measures were also examined, since mostly contradictory results have been reported to date (Rodrìguez-Fornells et al., 2002; Horn et al., 2003; Cheung et al., 2004; Spinella, 2004; Keilp et al., 2005; Lijffijt et al., 2005; Enticott et al., 2006; Reynolds et al., 2006; Aichert et al., 2012; Malesza and Ostaszewski, 2016). We aimed to clarify which aspects of trait impulsivity are related to response inhibition and which to reward responsiveness and hypothesized that the experimental tasks would significantly correlate with questionnaires measuring different aspects of impulsivity. This was met for the DDT, as there were significant positive correlations with the BIS-11 Total scale and the BIS-11 Non-Planning subscale. However, the rapid-response impulsivity tasks (GNGT, SST) did not directly correlate with any of the self-report measures. These results will be discussed in more detail in the following sections organized by impulsivity factors.

### Rapid-Response Impulsivity

The BIS-11 is one of the most widely used measures of trait impulsivity, and the Motor subscale assesses the tendency to act on the spur of the moment. This sub-scale examines the lack of inhibitory control observed in rapid-response impulsivity, the impulsivity dimension of interest here. In the current study, high rapid-response impulsivity group, as identified by their level of rapid-response impulsivity measured by the BIS-11 Motor subscale, showed significantly higher scores in all reward-delay impulsivity measures:

(1) Behaviorally, on the DDT, participants in high rapid-response impulsivity group preferred small but immediate rewards over larger, delayed rewards significantly more often than low rapid-response impulsivity group.
(2) On the BAS Total score, which measures BAS sensitivity toward rewards.
(3) On the BAS Fun Seeking subscale, which measures willingness to approach new and potentially rewarding experiences, and which is the only BAS subscale that overlaps with impulsivity (Alloy et al., 2009).
(4) On the UPPS (Lack of) Premeditation subscale, which measures the inability to reflect on the consequences of one’s actions before engaging in them, and which has been related to reward-delay impulsivity (Lynam and Miller, 2004).

Participants in the high rapid-response impulsivity group also had significantly higher scores on other motor impulsivity measures, such as the UPPS Urgency subscale, on measures theoretically related to rapid-response impulsivity, like the UPPS (Lack of) Perseverance subscale, and on trait impulsivity measures like the EPQ Psychoticism subscale. Contrary to our expectations, no significant differences were observed between participants in the high- and low-rapid-response impulsivity group on the GNGT and the SST tasks, which was surprising. Although high scores on the BIS-11 have been reported to be correlated with worse performance in the GNGT (e.g., Keilp et al., 2005; Enticott et al., 2006; Reynolds et al., 2006), other studies have reported that healthy individuals scoring high on self-report measures of impulsivity do not show impaired performance in response inhibition paradigms (Fallgatter and Herrmann, 2001; Horn et al., 2003; Dimoska and Johnstone, 2007; Lansbergen et al., 2007; Aichert et al., 2012; Wilbertz et al., 2014). This could be the case here too, as behavioral approaches have been suggested to measure task performance during a limited and at an exact moment in time, while self-report measures might focus on self-reported trait impulsivity, manifested across time and different situations (Lane et al., 2003; Reynolds et al., 2006; Cyders and Coskunpinar, 2011).

We also examined the associations between response inhibition paradigms and rapid-response impulsivity questionnaires. Contrary to our predictions, neither the GNGT nor the SST were significantly associated with self-report measures assessing rapid response impulsivity. Although previous studies have reported significant correlations between the GNGT and the BIS-11 (e.g., Spinella, 2004; Keilp et al., 2005; Enticott et al., 2006; Reynolds et al., 2006), others did not report such associations (e.g., Horn et al., 2003; Kulendran et al., 2016). Similarly, the SSRT, the main measure of the SST, has been shown to significantly correlate with the UPPS Sensation Seeking subscale (Aichert et al., 2012) and with the EPQ (e.g., Logan et al., 1997; Marsh et al., 2002). However, other studies have not reported such relationship (e.g., Rodrìguez-Fornells et al., 2002; Cheung et al., 2004; Keilp et al., 2005; Lijffijt et al., 2005; Enticott et al., 2006; Reynolds et al., 2006). It is possible that the fusion, or amalgamation of the different concepts of impulsivity has resulted in such inconsistencies (Cyders and Coskunpinar, 2011).

Taken together, our findings provide some evidence for a relationship between rapid-response and reward-delay impulsivity. Participants in the high rapid-response impulsivity group as measured by scores on the BIS-11 Motor subscale, showed significantly higher reward-delay impulsivity, both on the cognitive task, i.e., the DDT and self-report measures assessing reward sensitivity, i.e., the BIS/BAS. These findings are associated with medium to large effect sizes, despite modest sample sizes thus suggesting some robustness to the observations. We observed no associations between self-report measures and cognitive tasks measuring rapid-response impulsivity. Our results, therefore, suggest that the BIS-11 Motor subscale might not be assessing rapid-response impulsivity in the same way as the cognitive tasks assessing response inhibition. This may highlight the fact that self-report measures expected to specifically assess motor impulsivity (e.g., BIS-11 motor subscale, UPPS Urgency) might not directly be linked to the cognitive effects of rapid-response impulsivity. This finding is in line with other studies stating a lack of associations between cognitive tasks assessing impulsivity and trait measures of impulsiveness (e.g., Kulendran et al., 2016). As mentioned previously, task-based measures of impulsivity assess concepts like response inhibition at an exact moment in time and for a very short duration, while self-report measures focus more on trait impulsivity, expressed over time and different situations (Lane et al., 2003; Reynolds et al., 2006; Cyders and Coskunpinar, 2011). A lack of, or minimal correlations, between cognitive task of impulsivity and self-report measures have previously been argued to point toward the fact that these assessments may measure different aspects of impulsivity (e.g., Lane et al., 2003; Reynolds et al., 2006) or that this information is collected in different ways, and may be an assessment-related confound. Here, however, both self-report measures and cognitive tasks were conducted on a computer.

Furthermore, although Wilbertz et al. (2014) used BIS-11 Total scores to sub-divide their sample, they subsequently showed that the UPPS Urgency subscale, which measures “the tendency to engage in impulsive behaviors under conditions of negative affect” (Whiteside et al., 2005, p. 561) better explains individual variability in relation to RI performance. Although the UPPS Urgency subscale is conceptually similar to the BIS-11 Motor subscale, except for its focus on negative affect, it seems it may also be more sensitive to behavioral effects of rapid-response impulsivity.

### Reward-Delay Impulsivity

The BIS/BAS scales, specifically the BAS subscales, were used to classify participants by their level of reward-delay impulsivity (in line with Alloy et al., 2009). We compared participants scoring high on the BAS subscales (high reward-delay impulsivity group) to those with moderate scores (low reward-delay impulsivity group) on the same subscales. Because low BAS has previously been linked with unipolar depression (Fowles, 1988; Depue and Iacono, 1989; Kasch et al., 2002), the moderate BAS group was chosen for comparisons with the high BAS group. The reason for not using the specific BAS Reward Responsiveness subscale was based on a previous study which related this subscale to the responsiveness to already obtained rewards (Alloy et al., 2009), not to the expectation of receiving a reward. The high reward-delay impulsivity group showed significantly higher scores on the BIS-11 Motor subscale. Although no significant differences were found on the GNGT and SST, results on the BIS-11 Motor subscale suggest that this group also exhibits rapid-response impulsivity characteristics.

The high reward-delay impulsivity group also had significantly higher scores on measures related to trait impulsivity, such as the UPPS Sensation Seeking subscale. Consistent with previous findings (e.g., Alloy et al., 2006, 2009), our results indicate that participants with high reward-delay impulsivity show higher trait impulsivity AND higher rapid-response impulsivity, as measured by self-report measures. Likewise, those with high rapid-response impulsivity scored significantly higher on reward-delay impulsivity measures. These results, along with the significant correlations observed between the BIS-11 and all three BAS subscales, and between the DDT and the BIS-11 Total and BIS-11 Non-Planning subscale, suggest that rapid-response and reward-delay impulsivity are closely related to each other.

Importantly, the high reward-delay impulsivity group not only showed significantly higher trait impulsivity compared to the low reward-delay impulsivity group, but also performed significantly different on the reward-delay impulsivity task, the DDT. This, together with the medium to large effect size reported for this sample further validates the construct of reward-delay impulsivity as being a crucial characteristic of trait impulsivity. Previous studies using the BIS/BAS scales comparing task performance of patients and healthy individuals have reported significant differences for risk-taking (Black et al., 2014) or reward tasks, such as the card-sorting task (Hayden and Heilbronner, 2014), while others reported non-significant results when using paradigms assessing reward responsiveness (Alloy et al., 2012).

### Rapid-Response and Reward-Delay Impulsivity Combined

When combining both, the rapid-response impulsivity groups with the reward-delay impulsivity groups (see methods for detail), the group scoring high on both impulsivity dimensions and the group scoring low on both impulsivity dimensions, showed characteristics that were rather different to those of ‘single’ low vs. high-impulsive groups. The combined high rapid-response impulsivity and reward-delay impulsivity group performed significantly worse than the combined low rapid-response impulsivity and reward-delay impulsivity group on the two tasks measuring rapid-response impulsivity; they also preferred small but immediate rewards over larger, delayed rewards significantly more often. The latter indicates a more pronounced reward-delay impulsivity than that for the low rapid-response impulsivity and reward-delay impulsivity group.

While differences between high- and low-impulsivity groups are less clear when individuals are categorized based on either impulsivity dimension, differences between high- and low-impulsivity groups are more specific, and pronounced, when combining both dimensions. Although previous papers have not reported impaired performance in healthy individuals scoring high in trait impulsivity (e.g., Aichert et al., 2012; Wilbertz et al., 2014), our results suggest that self-report measures of rapid response impulsivity alone may not be sensitive enough to pick up the differences between groups in response inhibition paradigms (as seen in the current study, when using the BIS-11 Motor scores alone). However, when including a related, and perhaps necessary, dimension, namely that of reward-delay impulsivity, differences between groups were observed. However, the opposite could therefore also be true, i.e., our results also show that self-report measures of reward-delay impulsivity alone may not be sensitive enough to pick up the differences in response inhibition paradigms. However, when including a related, and perhaps necessary, dimension, namely that of rapid-response impulsivity, differences between groups were observed. These findings should be taken with caution, however, as the number of individuals in this combined impulsivity group is quite low, as only about 13.8% of participants scored high on both dimensions of impulsivity.

Additional analyses were therefore conducted to assess whether the differences in results between combined impulsivity groups and single impulsivity groups were affected by the difference in participant numbers. This would indicate whether the observed effects were due to comparing the very extreme ends of impulsivity or were truly related to the combination of the two impulsivity dimensions. To test this, separate MANCOVAs were performed for rapid-response and reward-delay impulsivity including only the top 22 low- and the top 23 high-scorers, i.e., the same number of individuals in each group as for the combined impulsivity group analysis. MANCOVAs showed that groups were not significantly different when classified by level of reward-delay impulsivity (Wilks λ = 0.86, *F*(5,38) = 1.28, *p* > 0.05) or by level of rapid-response impulsivity (Wilks λ = 0.90, *F*(5,38) = 0.87, *p* > 0.05). These analyses showed no significant differences between groups in any of the three cognitive tasks. Therefore, it is the combination of the two dimensions of impulsivity that provides a more sensitive assessment.

### Limitations

While the high reward-delay impulsivity group made significantly more commission errors on the GNGT, we expected the SST would show similar results, as both tasks are thought to measure rapid-response impulsivity. The lack of significant results on the SST in this specific comparison might be explained by the absence of a staircase procedure, which would have adapted the time between the “go” and “stop” stimuli based on whether the previous trial was successfully inhibited or not, increasing the difficulty of the task. Current findings should be taken with caution, as the number of individuals in the group which combined the high rapid-response impulsivity individuals and the high reward-sensitivity individuals is quite low. Only about 13.8% of participants scored high on both dimensions of impulsivity, which limits the generalizability of our data. However, Wilbertz et al. (2014) who used the BIS-11 to identify high or low impulsive individuals reported selecting 52 from 452 participants which is a similar percentage. Our participants were recruited entirely from a student population and findings may thus not be generalizable to other populations. Although GNG tasks have widely been used within neuropsychological assessments (Luria, 1973; Drewe, 1975), limited information regarding their standardization and validation appears available (Langenecker et al., 2007). The GNGT’s internal validity is thought to be limited because there are many variants of the task (Bodnar et al., 2007). Furthermore, GNG tasks vary greatly in terms of proportion of go and no-go stimuli, the types and timing of the stimuli used and in how similar target and non-target stimuli can be (Langenecker et al., 2007). Our study uses letters, in both the GNGT and SST, stimuli which may be less ecologically valid than using pictorial images, for example faces, in response inhibition tasks. Schulz et al. (2007), however, showed that commission errors on a traditional GNGT task (using circles) and those on an emotional GNGT (using facial expressions) showed moderate correlations between both measures. Further limitations that should be considered are limited screening for psychopathology, no screening for neurological conditions or use of prescription medication taken at the time of testing. Any of these factors could affect the presented results.

### Implications and Future Directions

Here, we have shown that the combination of different dimensions of impulsivity is better able to differentiate individuals who present high and low levels of trait impulsivity. Given that impulsivity has been reported to characterize several mental disorders it is feasible to investigate impulsivity in the context of mental disorder, and to consider its potential to predict risk for psychopathology. It seems plausible that reward-delay impulsivity and response inhibition represent shared vulnerabilities for mental health conditions characterized by deficits in impulse control, e.g., bipolar disorder, substance use disorders and addictions (e.g., Bari and Robbins, 2013). Furthermore, both, Diagnostic and Statistical Manual of Mental Disorders (DSM) and International Classification of Diseases (ICD), recognize impulsivity as a diagnostic criterion for these disorders, and specifically those aspects which can be considered unplanned, on-the-spur-of-the-moment reaction with no regard for consequences, sense of urgency and self-harming behaviors, especially during times of emotional instability (Moeller et al., 2001). Therefore, it is important to investigate these two dimensions in conjunction.

Furthermore, given the behavioral and psychometrical base of measures of rapid response and reward-delay impulsivity, they might be used in future to aid in designing a screening tool to assess risk for psychopathology based on these two impulsivity dimensions. Such a screening tool could also benefit from the clarification of personality characteristics provided here, on each dimension of impulsivity. The acknowledgment of the specific aspects of impulsivity could also be useful when providing individuals high in impulsivity with strategies to recognize impulsive behaviors or thoughts, or with tools to reduce their impulsivity in certain situations, as in mindfulness-based strategies to reduce impulsivity (e.g., Stratton, 2006; Lattimore et al., 2011). Furthermore, neuroimaging studies using these response inhibition and delay discounting paradigms could compare high and low impulsive groups and provide detailed information of the neural correlates of these impulsivity dimensions in young healthy individuals.

## Conclusion

In summary, this study compared different measures of impulsivity, to examine how sensitive these measures are to differences between low and high impulsive groups, based on two impulsivity dimensions: rapid-response impulsivity and reward-delay impulsivity. Results show that the proposed measures were sensitive to differences between groups. Participants with higher impulsivity, as measured by high rapid-response impulsivity scores on the BIS-11 Motor subscale, show significantly increased trait impulsivity as well as behavioral and self-reported reward-delay impulsivity. Conversely, the high reward-delay impulsivity group had significantly higher trait impulsivity and behavioral and self-reported rapid-response impulsivity. When both dimensions of impulsivity were combined, the high-impulsivity group performed significantly worse on both response inhibition paradigms (GNG and SST) and temporally discounted in a significantly more impulsive manner in the reward-delay task than the low-risk group. These findings provide evidence that combining impulsivity dimensions provides a better predictor of impulsivity level than each dimension alone.

## Author Contributions

AJ and SH have contributed to this study in conception and design, and in the collection and analysis of data. All authors were involved in the interpretation of results and in drafting and revising the manuscript. All authors have given it their approval.

## Conflict of Interest Statement

The authors declare that the research was conducted in the absence of any commercial or financial relationships that could be construed as a potential conflict of interest.

## Footnotes

1 http://www.pstnet.com/

2 http://www.onlinesurveys.ac.uk
